# An alternative method for personalized tourniquet pressure in total knee arthroplasty: a prospective randomized and controlled study

**DOI:** 10.1038/s41598-022-13672-6

**Published:** 2022-06-10

**Authors:** Jun Wu, QiWei Fu, HaoBo Li, YaGuang Han, JianHua Deng, Yi Chen, QiRong Qian

**Affiliations:** 1 Department of Joint Surgery and Sports Medicine, Changzheng Hospital, Naval Medical University, 415#, Fengyang Road, Huangpu District, Shanghai, 200003 China; 2 Department of Orthopaedic Surgery, Nantong Hospital Affiliated to Shanghai University (Nantong Sixth People’s Hospital), 500#, Yonghe Road, Chongchuan District, Nantong, Jiangsu Province 226011 China

**Keywords:** Diseases, Trauma, Skeleton, Bone

## Abstract

Tourniquet use always carries potential risks, which can range from mild transient functional impairments of thigh pain, skin blisters to severe permanent dysfunction of limb paralysis, nerve injuries or compartment syndrome. The ideal method for minimizing intraoperative tourniquet pressure (TP) for reducing postoperative complications remains controversial. In this prospective, randomized and controlled study, we reinvestigated an estimation formula for TP based on thigh circumferences and systolic blood pressure (SBP) with two traditional methods for TP determination in total knee arthroplasty (TKA): SBP plus 100 mmHg and a fixed value of 300 mmHg. TP values and postoperative thigh pain scores were compared among three groups. The intraoperative TP value of the formula-calculated group was lower than that of the traditional groups (14.7 mmHg, *P* = 0.3475 and 94.7 mmHg, *P* < 0.0001, respectively), while no differences of hemostatic effect at the surgical fields and wound complications were detected among groups. The thigh pain scores at the tourniquet site decreased gradually over time and the estimation group had the lowest scores at each timepoint after surgery. Estimation method for TP was easy and rapid, without relying on specific equipment. It could provide a practical low TP and comparable hemostatic effect in TKA using an inflating tourniquet.

## Introduction

In total knee arthroplasty (TKA), tourniquet use has become a standard procedure to reduce blood loss and obtain a bloodless surgical field for soft tissue resections, osteotomies, and the implantation of prostheses^1,2^. However, tourniquets use always carries potential risk of nerve and soft tissue injuries, which can range from mild transient functional impairments including postoperative thigh pain, skin blisters and damage to subcutaneous tissue^3^, to severe permanent dysfunction including rhabdomyolysis^4^, limb paralysis^5^, nerves and injuries^2,6^, ischemic or thromboembolic complications^7,8^, and compartment syndrome^9,10^. In more than 70% of lower limb surgeries, nerve conduction impairment and muscle dysfunction have been documented, and these complications are underreported in clinical practice^11^. Evidence suggests that these potential complications are mostly attributable to excessive tourniquet pressure (TP) and time. Anyhow, these incidences can be reduced by minimizing tourniquet time and cuff pressure as much as possible to the level of artery occlusion pressure (AOP)^2,8,12^.

In clinical practice, there is no universally accepted standard to identify the optimal TP. For lower limb surgery, some surgeons may routinely choose 250 or 300 mmHg^13^, while most set it at the level of systolic blood pressure (SBP) plus 100 mmHg^14^. However, these methods do not take other individual differences into account. Limb AOP is the minimal pressure that is required, using a specific tourniquet cuff at a specific location on the limb, to occlude the arterial blood flow into the distal part of an individual limb^15,16^. The actual TP before surgery is AOP plus a safety margin to cope with intraoperative fluctuation of SBP^16^. However, AOP measurement usually relies on specific tourniquet apparatus and the high costs limit wide application. Tuncali et al.^17^ reported an innovative formula for estimating AOP based on the tissue padding coefficient (K_TP_) and SBP: AOP = (*SBP* + 10 mmHg)/*K*_*TP*_, which was shown to be simple, without requiring any special equipment^18,19^.

This study aimed to evaluate the safety and feasibility of the estimation method mentioned above in identifying patients’ individual TP, compared to the conventional methods in clinical practice. We hypothesized that the estimation group had lower TP value and visual analog scale (VAS) score for postoperative thigh pain than that in the conventional groups, while without compromise to the bloodless quality of the surgical filed, and postoperative complications would not be substantially different.

## Results

During the recruitment period, from January 2019 to November 2019, 181 patients with unilateral knee osteoarthritis scheduled to get TKA in our department were assessed for eligibility. 31 patients were excluded for the following reasons: 11 patients had type-2 diabetes mellitus, one obese patient with body mass index (BMI) = 31.6 kg/m^2^, and 19 patients refused to take part in this trial. A total of 138 patients in this study completed at least 3 months of follow-up: 46, 45 and 47 patients in groups A and B, respectively, were included in the final analysis. 12 patients did not complete the 3-month follow-up because of migration and were excluded from the analysis.

Females accounted for 62.7% (94/150) of all participants. The mean age was 71.2 ± 6.2 years, mean BMI was 23.8 ± 2.8 kg/m^2^ and the mean thigh circumference was 49.7 ± 7.1 cm. Among the three groups, baseline characteristics were comparable. No differences were observed regarding sex, age, BMI, circumference of the thigh, preoperative knee pain, preoperative SBP, American Society of Anesthesiologists (ASA) class, and severity of osteoarthritis (Kellgren-Lawrence Classification of Osteoarthritis) between groups (Table 1).

**Table 1:** Demographic and interoperation data among groups.

Baseline data	Group A	Group B	Group C	*P* value
Number of patients (male/female)	50 (19/31)	50 (18/32)	50 (19/31)	0.9719
Age (yr)	71.7 ± 6.1	70.3 ± 6.2	71.7 ± 6.3	0.4296
BMI (Kg/m^2^)	24.0 ± 2.7	23.9 ± 2.9	23.6 ± 2.6	0.7491
Thigh Circumference (cm)	49.2 ± 6.5	50.3 ± 8.6	49.7 ± 6.1	0.7629
Knee pain (VAS Scores)	5.9 ± 1.1	5.8 ± 1.3	6.0 ± 1.1	0.4564
Knee range of motion (degree)	93.9 ± 12.6	94.4 ± 13.2	93.5 ± 8.9	0.9340
Kellgren–Lawrence (III/IV)	26/24	30/20	26/24	0.6502
ASA (I/II/III)	26/18/6	33/16/1	28/19/3	0.2914

Significant values are in italics. BMI, body mass index. VAS, visual analog scale. Kellgren-Lawrence, Kellgren-Lawrence Classification of Osteoarthritis. ASA, American Society of Anesthesiology.

**Figure 1:**
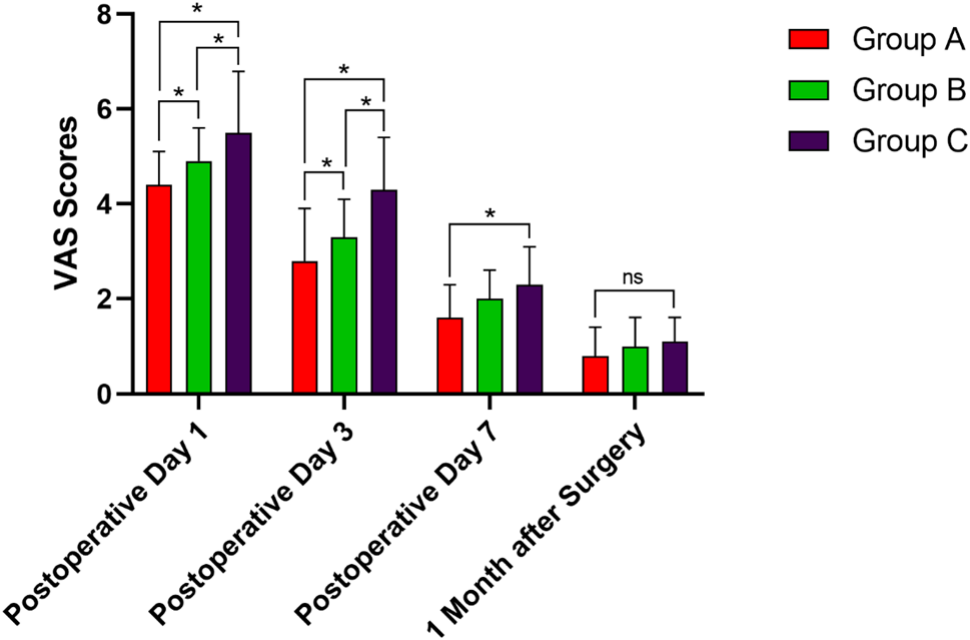
Differences of postoperative thigh pain VAS (visual analog scale) scores among groups. *represents the difference between these two groups was statistically significant (*P* < 0.05); ns, represents no significant inter-group difference was observed.

**Figure 2:**
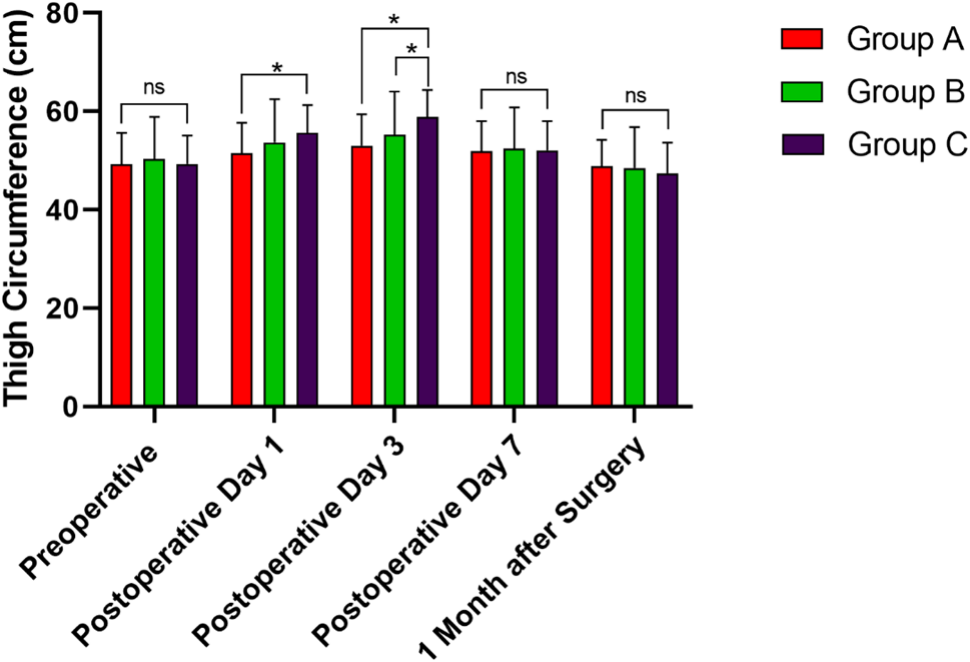
Differences of pre-. and postoperative thigh circumferences (cm) among groups. *represents the difference between these two groups was statistically significant (*P* < 0.05); ns, represents no significant inter-group difference was observed.

**Figure 3:**
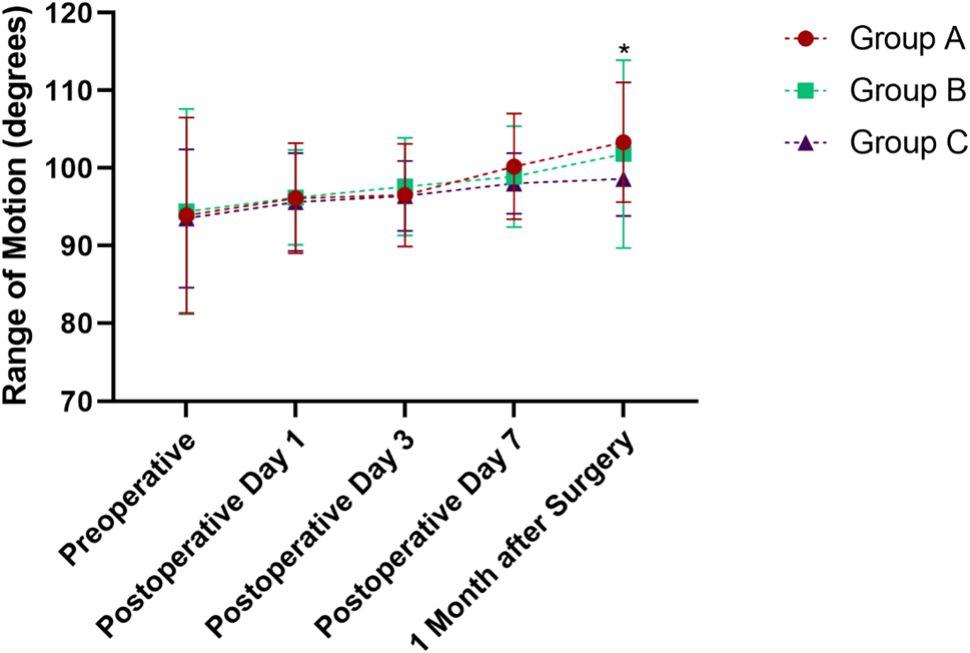
Differences of postoperative knee joint ROM (range of motion, degree) among groups. *represents the difference between groups A and C was statistically significant (*P* < 0.05).

In group A, the time required to determine TP was 8.6 ± 1.2 min, including measuring and calculating. As shown in Table 2, compared with groups B and C, group A had a lower mean TP of 205.3 ± 86.3 mmHg, with a difference of 14.7 mmHg (95% CI − 10.31 to 39.71, *P* = 0.3475) and 94.7 mmHg (95% CI 69.96–119.4, *P* < 0.0001), respectively. In group B, a significantly lower TP of 220.0 ± 12.5 mmHg was recorded compared to that of group C (95% CI 55.12–104.9, *P* < 0.0001). The thigh pain score at the tourniquet site decreased gradually over time postoperatively. On day 1 and day 3, significant differences were observed among groups. On day 7, significant difference only existed between groups A and C (*P* = 0.0002, Fig. 1). The postoperative thigh circumference of each group was the largest on the third day after surgery: when groups A (53.0 ± 6.4 cm) and B (55.3 ± 8.7 cm) was significantly smaller than that of group C (58.8 ± 5.6 cm) (*P* = 0.0002 and 0.0414, respectively). On the first day after surgery, the significance only existed between groups A and C (*P* = 0.0124, Fig. 2). The quality of the bloodless surgical field rating was satisfied in all groups (mean, 9.6 ± 0.6, 9.7 ± 0.6, 9.8 ± 0.4, respectively), while no significant inter-group difference was detected (*P* = 0.0226). The knee range of motion (ROM) increased on time, and significant difference only existed on 1 month after surgery (*P* = 0.0137, Fig. 3).

**Table 2:** Comparison of outcomes among groups.

	Group A	Group B	Group C	*P* value
**Cuff pressure and surgeon rating of bloodless surgical field**				
Systolic blood pressure preoperative (mmHg)	121.0 ± 17.2	120.5 ± 12.7	119.0 ± 11.4	0.7729
Tourniquet pressure (mmHg)	205.3 ± 86.3^a^	220.0 ± 12.57^b^	300.0 ± 0.0^a,b^	*< 0.0001*
Tourniquet time (min)	77.0 ± 2.0	76.9 ± 2.8	76.0 ± 7.0	0.5055
Bloodless surgical Field assessment (*VAS scores*)	9.6 ± 0.6	9.7 ± 0.6	9.8 ± 0.4	0.2071
**Tourniquet related complications**				
Postoperative thigh pain (*VAS scores*)				
Day 1	4.4 ± 0.5*	4.9 ± 0.6*	6.5 ± 1.4*	*< 0.0001*
Day 3	2.8 ± 1.1*	3.3 ± 0.8*	4.3 ± 1.1*	*< 0.0001*
Day 7	1.6 ± 0.7^c^	2.0 ± 0.6	2.3 ± 0.8^c^	*< 0.0001*
1 month	0.8 ± 0.6	1.0 ± 0.6	1.1 ± 0.5	0.7958
Postoperative thigh circumference (cm)				
Day 1	55.5 ± 6.2^d^	53.7 ± 8.8	55.6 ± 5.7^d^	*< 0.0001*
Day 3	53.0 ± 6.4^e^	55.3 ± 8.7^f^	58.8 ± 5.6^e, f^	*< 0.0001*
Day 7	51.9 ± 6.1	52.4 ± 8.4	52.1 ± 5.9	0.9409
1 month	48.8 ± 5.4	48.5 ± 8.3	47.4 ± 6.2	0.5732
Skin Blister	0	0	2	–
Limb paralysis/paresthesia	0	0	0	–
Wound complications (delayed healing/infection)	0	0	0	–
**Knee joint function after surgery**				
ROM postoperative (degree)				
Day 1	96.1 ± 7.1	96.2 ± 6.1	95.6 ± 6.3	0.8933
Day 3	96.5 ± 6.6	97.6 ± 6.3	96.4 ± 4.5	0.5571
Day 7	100.2 ± 6.8	98.9 ± 6.5	98.0 ± 3.9	0.1954
1 month	103.3 ± 7.7^g^	101.8 ± 12.1	98.6 ± 4.8^g^	*< 0.0001*
Knee society score (third month after surgery)				
KSS-C	88.7 ± 3.4	87.6 ± 3.5	88.3 ± 3.1	0.2852
KSS-F	86.6 ± 3.8	85.0 ± 3.4	85.3 ± 3.5	0.0782

Significant values are in italics. *represents the differences among three groups was statistically significant (P<0.05). Any two values with the same or containing one same letter superscript, means that there is statistical difference between these two groups.

Small blisters were observed in 2 patients in group C immediately after tourniquet deflation, but there was no enough statistical power to detect an intergroup difference. No newly formed blisters or skin necrosis were observed after surgery in any patient. No complaints of paralysis or paresthesia were reported in either group before or after discharge. No wound complications were recorded at suture removal or during the follow-up visits.

At the follow-up on postoperative month3, the mean clinical Knee Society Score (KSS, KSS-C) was 88.7 ± 3.4, 87.6 ± 3.5, and 88.3 ± 3.1in groups A, B, and C, respectively. The functional KSS (KSS-F) was 86.6 ± 3.8, 85.0 ± 3.4, and 85.3 ± 3.5 in groups A, B, and C, respectively. The differences between the three groups were not statistically significant (KSS-C, *P* = 0.2852 and KSS-F, *P* = 0.0782).

## Discussion

In this study, formula-based estimation of personalized AOP was simple and fast to calculate. In the estimation group A, the TP value was generally lower than SBP plus 100 mmHg in group B and significantly lower than 300 mmHg in group C, while providing comparable quality of the bloodless surgical field. Further, group A with lower TP had significantly lower thigh pain scores than the groups B and C on postoperative days 1 and 3, and did than group C on postoperative day 7. The generally lower cuff pressure in the estimation group did not induce any additional risk of postoperative complications. These results confirmed our hypothesis on the efficacy and safety of the estimation method.

The AOP determination based on Doppler-ultrasound techniques is considered to be the “golden standard”. However, these methods highly rely on additional operator skills, ultrasound equipment, or a tourniquet system with specific functions and are time consuming, which largely limits their use at primary hospitals in developing countries and areas^20–23^. Cadaver and animal studies found that tissue pressure underneath the tourniquet cuff was lower than the superficial layers and decreased towards the artery at the center of the limb, correlated with limb circumference and shape^24,25^. Tuncali^17^ introduced an estimating formula based on a patient’s thigh circumference and SBP: AOP = (*SBP* + 10 mmHg)/*K*_*TP*_. In this study, Tuncali and his colleagues measured the tissue pressure under the tourniquet (TPUT) and tourniquet inflation pressure (TIP) and developed a regression model of TPUT expressed as a percentage of TIP (*K*_*TP*_) versus limb circumference: TPUT = 1.876 × [Extremity circumference (cm) ^−0.2399^]. Kasem et al.^19^ found this formula was more effective in estimating the lowest effective TP in surgeries of the lower limbs than the estimating formula proposed by Liu et al.^26^.

According to the fast-track surgery protocol in TKA, peri-joint pain after surgery is one of the most important factors affecting the early rehabilitation and postoperative knee function^27^. As shown in the Fig. 1, groups with lower TP had lower thigh pain score at each time-point, especially on day 1 and 3 after surgery, indicating that TP was an important cause of thigh pain. Caparrini et al.^28^ adopted a combination of multimodal pain management protocol in TKA and all the patients could walk with the aid of two crutches on the second postoperative day. The thigh circumference at the tourniquet site is another indicator of local soft tissue injury^5^. In the Fig. 2, the mean thigh circumference in each group was the largest on the third day after surgery, when inter-group statistical significance was detected. Unver et al.^29^ found that tourniquet application with lower pressure can gain more rapidly early functional mobility. In our study, although the knee ROM was better in group A than that in group C 1 month after surgery, we could not be able to draw a firm conclusion about the association between postoperative knee ROM and TP. Similarly, Alexandersson and his colleagues compared two groups of TKA patients with or without tourniquet and found that postoperative mobility improvement between two groups was not at a clinically relevant level^30^.

Regardless of the method used, the hemostatic effect was excellent in all three intervention groups. The estimation group provided comparable bloodless surgical fields with a relatively lower pressure. Kasem et al.^19^ used the method and found that good bloodless surgical fields could be achieved at a TP of 208 ± 12 mmHg. Kim et al.^31^ compared the low cuff pressures of SBP + 120 mmHg to higher ones of SBP + 150 mmHg and found no difference in the quality of the bloodless surgical field, safety outcomes, and tourniquet-related complications. We conclude that a lower tourniquet pressure can be used effectively in TKA with satisfactory bloodless surgical fields.

SBP is a controllable factor that directly affects the intraoperative AOP and is unlikely to remain motionless. In Tuncali’s study^32^, the mean TP value in AOP group was 160.04 ± 14.17 mmHg with an SBP of 105.67 m ± 9.81 mmHg while Olivecrona et al.^33^ reported a higher mean TP value of 246 ± 45 mmHg with an SBP of 155 ± 21 mmHg. To obtain a low TP, SBP should be managed to be as low as possible and maintained as stable as possible during surgery^34^, especially in obese patients^35,36^. This estimation method had one disadvantage that SBP had to be monitored every 10 min with corresponding TP adjustments intraoperatively. However, it was precisely for this reason that intraoperative TP was more accurate and much closer to the actual AOP than the fixed values in other groups.

Except for records of small blisters in 2 patients in group C after tourniquet deflation, there was no soft tissue or incision complication observed after surgery in any patient. However, Olivecrona et al.^33^ reported a higher wound complication rate than that in our study, which might result from their inclusion of patients with diabetes. Clarke et al.^37^ studied postoperative hypoxia of the skin flap and they found that fewer incision complications occurred when the TP pressure was lower than 225 mmHg, indicating that this value maybe the threshold above which postoperative incision complications become more likely. TP should always be chose as low as possible unless the satisfied bloodless surgical field could not be achieved or prosthesis implantation was affected.

This study had several limitations. Firstly, the reliability of AOP in the measurement groups had not been verify because of time constraints in operation room. Secondly, general anesthesia was used in all patients in our study. However, many institutions use intravertebral anesthesia for TKA and as a result, SBP tends to fluctuate greatly during surgery. In this case, the reliability of the estimation method needs to be further investigated. Thirdly, VAS scores were assessed by only one senior surgeon, the observer reliability needs to be validated. Finally, because of the number of patients, this study was underpowered to draw any meaningful conclusion regarding postoperative complications. Multicenter prospective studies with a larger number of patients can help address these issues and verify the conclusion of the present study.

## Methods

### Study design

This prospective randomized controlled study was registered in the Chinese Clinical Trial Registry (20/01/2019, ChiCTR1900020840) and conducted in accordance with the Consolidated Standards of Reporting Trials (CONSORT) statement. Research approval was obtained from the Ethics Committee of Changzheng Hospital Affiliated to Naval Medical University (Approval No. 2018SL51), and written informed consents were obtained from all patients. All methods used in this study were performed in accordance with the “Guidelines and Regulations of Clinical Study” in Shanghai Changzheng Hospital. All patients underwent TKA at our institute from January 2019 were eligible for this study.

Inclusion and Exclusion criteria:

Inclusion criteria were as follows: patients with (1) unilateral degenerative osteoarthritis of the knee consistent with the diagnostic criteria for knee osteoarthritis (ICD-10 M17.901); (2) normal muscle strength and limb sensation; and (3) normal hemoglobin (Hb) levels (120 g/L ≤ Hb ≤ 160 g/L in males and 110 g/L ≤ Hb ≤ 150 g/L in females), coagulation and renal function.

Exclusion criteria included: patients with (1) diabetes mellitus; (2) neuromuscular or vascular disease; (3) mental disease disturbing functional rehabilitation and follow-up visits; (4) BMI > 30 kg/m^2^; (5) total knee revision surgery; and (6) unstable concurrent chronic disease, if any.

**Figure 4:**
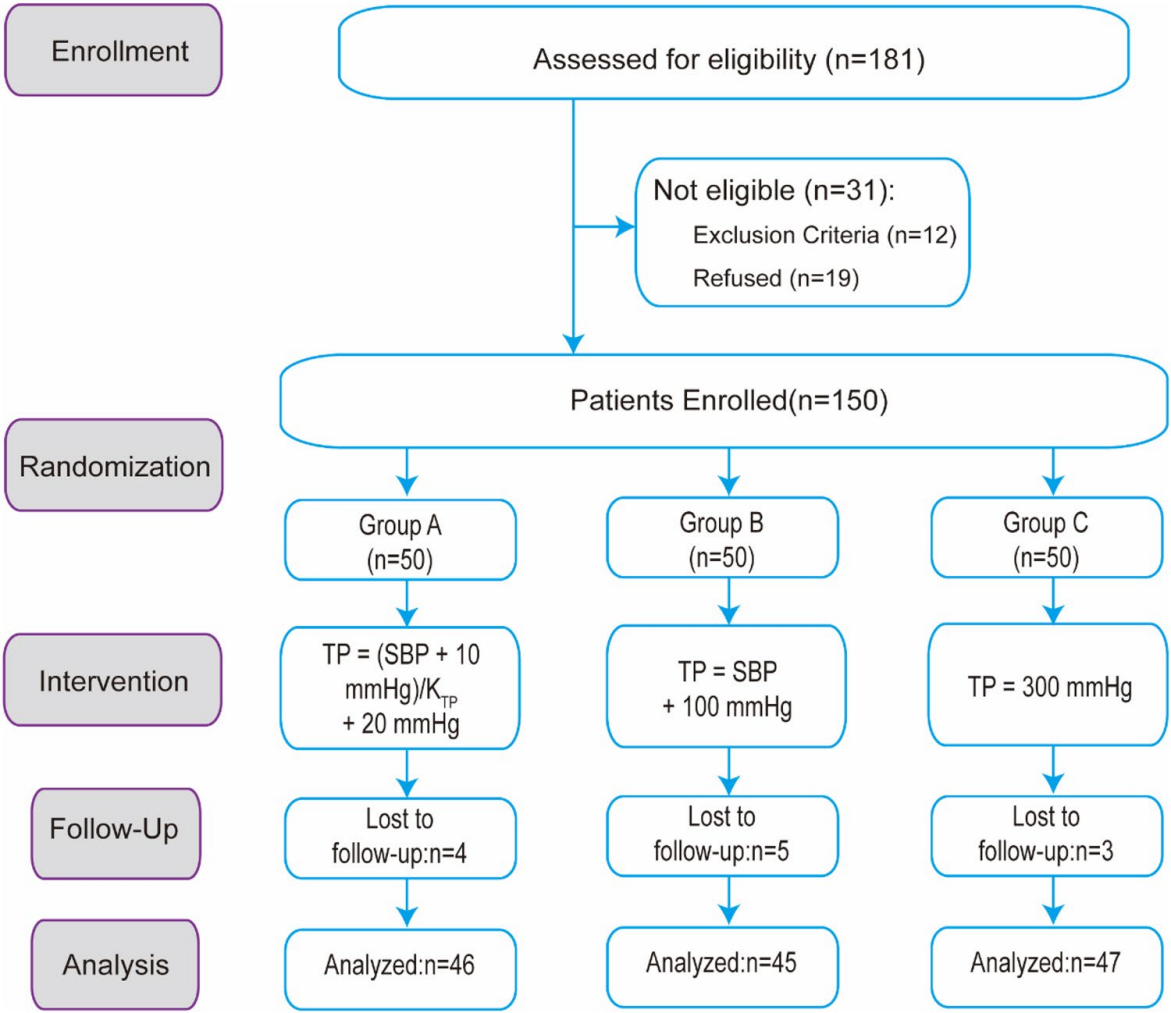
CONSORT (Consolidated Standards of Reporting Trials) flow diagram of patients eligible and intervention of this study. TP, tourniquet pressure. SBP, systolic blood pressure.

The CONSORT diagram for this trail is shown in Fig. 4.

### Patients recruitment and intervention

A total of 150 patients who underwent primary TKA were enrolled in this study from Jan. 2019 to Nov. 2019. Patients were randomly allocated to three intervention groups using Microsoft Excel random-number-generator function (version 2016, Microsoft Corporation, Redmond, WA, US): 50 patients each were assigned to group A, B and C. Researchers who designed the study generated the random allocation, enrolled and assigned participants into intervention. Grouping information of each patient was sealed in an envelope till surgery.

In group A, AOP was calculated based on the formula AOP = (*SBP* + 10 mmHg)/*K*_*TP*_^17^. The corresponding relationship between the coefficient *K*_*TP*_ and limb circumference is shown in Table 3. Thigh circumference was measured 20 cm proximal to the upper pole of the patella with the knee fully extended. Inflating TP was determined by adding a safety margin of 20 mmHg to AOP to ensure intraoperative artery occlusion was complete in case of SBP fluctuation. SBP measurements were performed in 10-min intervals, and the TP was adjusted by 10-mmHg for every 10-mmHg fluctuation of SBP. In group B, SBP of each patient was measured and recorded 10 min after general anesthesia when SBP was stable. The TP value was determined at the level of SBP plus a safety margin of 100 mmHg. In group C, TP of each patient was set at the fixed value of 300 mmHg. No intraoperative adjustments of TP were made in groups B and C unless the surgeon was not satisfied with the bloodless surgical field or the operation could not be continued.

**Table 3:** Tissue padding coefficient (K_*TP*_) adjusted to limb circumferences^17^.

Extremity circumferences (cm)	Estimated *K*_*TP*_
20	0.91
21	0.90
22	0.89
23	0.88
24	0.87
25	0.86
26 to 27	0.85
28	0.84
29	0.83
30 to 31	0.82
32 to 33	0.81
34	0.80
35 to 36	0.79
37 to 38	0.78
39 to 40	0.77
41 to 43	0.76
44 to 45	0.75
46 to 48	0.74
49 to 51	0.73
52 to 54	0.72
55 to 57	0.71
58 to 60	0.70
61 to 64	0.69
65 to 68	0.68
69 to 73	0.67
74 to 75	0.66

In all groups, ATS® 3000 tourniquet system (Zimmer Surgical, Inc, Ohio, US) and an 11-cm wide double-bladder tourniquet was used with its distal edge placed 15 cm above the superior pole of the patella. Sex, age, BMI, the severity of osteoarthritis (Kellgren–Lawrence Classification of Osteoarthritis), SBP, and surgical and tourniquet time were recorded.

### Surgical procedure and perioperative management

All patients undergoing TKA received standardized perioperative care, including health assessment, surgical team, nurse care, and operating room personnel. General anesthesia was adopted. Second-generation cephalosporin antibiotics were administered for prophylaxis of infection 30 min before skin incision. Medial parapatellar approach and a cemented posterior-stabilized prosthesis (NexGen® LPS; Zimmer Biomet, Warsaw, IN, US) was applied for TKA by the same experienced senior surgeon. Postoperative management followed the Expert consensus in enhanced recovery after total hip and knee arthroplasty in China: perioperative management^38^.

All patients, surgeons, ward nurses, and researchers who carried out follow-up visits and data collection were blinded to the patients’ group. The nurses who unsealed the grouping envelope, estimated and set the TP in the operating room did not participate in postoperative care, follow-up visits, or data analysis.

### Outcome measures

Primary outcome measures were the average intraoperative TP value and the thigh pain on postoperative day 1, 3, 7 and 30. Secondary outcome measures were the bloodless effect in the surgical field, thigh circumference and knee joint ROM at different postoperative time-point (postoperative day 1, 3, 7 and 30), wound complications, and the Knee Society Score (KSS) at the third-month follow-up visit.

The senior surgeon who was in charge of the operation rated the quality of the bloodless surgical field immediately after surgery, using a visual analog scale (VAS), with a score of 10 indicating the highest satisfaction and a score of 1 indicating the lowest. The skin underneath the tourniquet cuff was examined for bruise, blisters, and necrosis immediately after surgery. On postoperative day 1, 3, 7 and 30, patients were required to rate their thigh pain via VAS. Thigh circumferential and knee joint ROM was measured both at in- and out-patient department on the above-mentioned four postoperative time-points by experienced researchers. Wound complications were recorded until the stitches were removed. At the third-month follow-up visit, KSS was used to assess knee pain, stability, and function in each patient.

### Data analysis and sample size calculation

Prism software, Version 8.0.2 (GraphPad Software, Inc., San Diego, CA, US) was used to analyze the results. Prior to data analysis, the distribution of potential confounders between groups as well as the primary and secondary outcomes were evaluated with summary statistics, including the mean and standard deviation for normally distributed quantitative data and the percentage for qualitative data. Binomial data are presented as the number and percentage. *One-way* analysis of variance was used for comparisons of continuous variables between groups. Non-normally distributed quantitative data were compared using the *rank-sum* tests. The *Chi-square* test was applied to compare differences in the categorical data between groups. A P-value < 0.05 was considered to be statistically significant.

We calculated the sample size using PASS 2011 software (NCSS, LLC, Kaysville, UT, USA) based on 80% power at a 5% significance level. According to the previous studies, we assumed the difference of the TP value was 50 mmHg between groups, and 41 patients were required in each group. The recruitment goal was set at 150 patients, anticipating a drop-out rate of about 20%.

## Conclusion

The formula-based estimation method with lower TP value was simple and provided comparable hemostatic effect to conventional methods. In lower extremity surgery using AOP technology, the estimation method could be a practical alternative worthy of promotion without specific apparatus.

## Data Availability

All data and materials are contained within the manuscript.
